# Supplementary material for: Lyl-1 regulates primitive macrophages and microglia development
Source: Commun Biol. 2021 Dec 9;4:1382. doi: 10.1038/s42003-021-02886-5 (PMC8660792; doi:10.1038/s42003-021-02886-5)
Supplement: Supplementary file 2 — Supplementary Information [file 42003_2021_2886_MOESM2_ESM.pdf]

Lyl-1 regulates primitive macrophages and microglia development

Supplementary information

Shoutang Wang[1, †, *], Deshan Ren[1, §, *], Brahim Arkoun[1], Anna-Lila Kaushik[1, #], Gabriel Matherat[1, $], Yann Lécluse[2], Dominik Filipp[3], William Vainchenker[1], Hana Raslova[1], Isabelle Plo[1], Isabelle Godin[1]

[1] Gustave Roussy, INSERM UMR1287, Villejuif; Université Paris-Saclay, France.

[2] PFIC, lUMS AMMICa (US 23 INSERM / UMS 3655 CNRS; Gustave Roussy, Villejuif, France

[3] Laboratory of Immunobiology, Institute of Molecular Genetics of the Czech Academy of Sciences, Prague, Czech Republic.

Supplementary note 1, related to Figure 1c-e, 3a and Supplementary figure 2a-d

During YS development, both $M\Phi^{Prim}$ and $M\Phi^{T\text{-}Def}$ progenitors originate from $cKit^+CD31^+CD45^-$ progenitors (C subset), which differentiate into MΦs via 3 subsets (A1 to A3)[1] ***(Supplementary figure 2a)***. In *Lyl-1$^{LacZ/LacZ}$* E9-YS, a large $CD11b^+CD31^+CD45^{neg/low}$ subset was identified through flow cytometry analyses of the distribution of MΦ progenitor subsets. This $CD11b^+CD31^+CD45^{neg/low}$ subset was nearly absent from WT E9-YS and it disappeared from mutant YS after E9.5 ***(Supplementary figure 2c)***. This transient subset, which display a phenotype intermediate between the C and A1 subsets, was also characterized by the expression of Lyl-1, as shown using Facs-Gal assay ***(Supplementary figure 2d)***. Together with the increased production of $M\Phi^{Prim}$ progenitors observed in clonogenic assays in mutant E8-YS (***Figure 1c)***, this observation suggests that in a WT context, Lyl-1 might negatively regulate the commitment, expansion and/or timing of production of $M\Phi^{Prim}$.

Supplementary figures

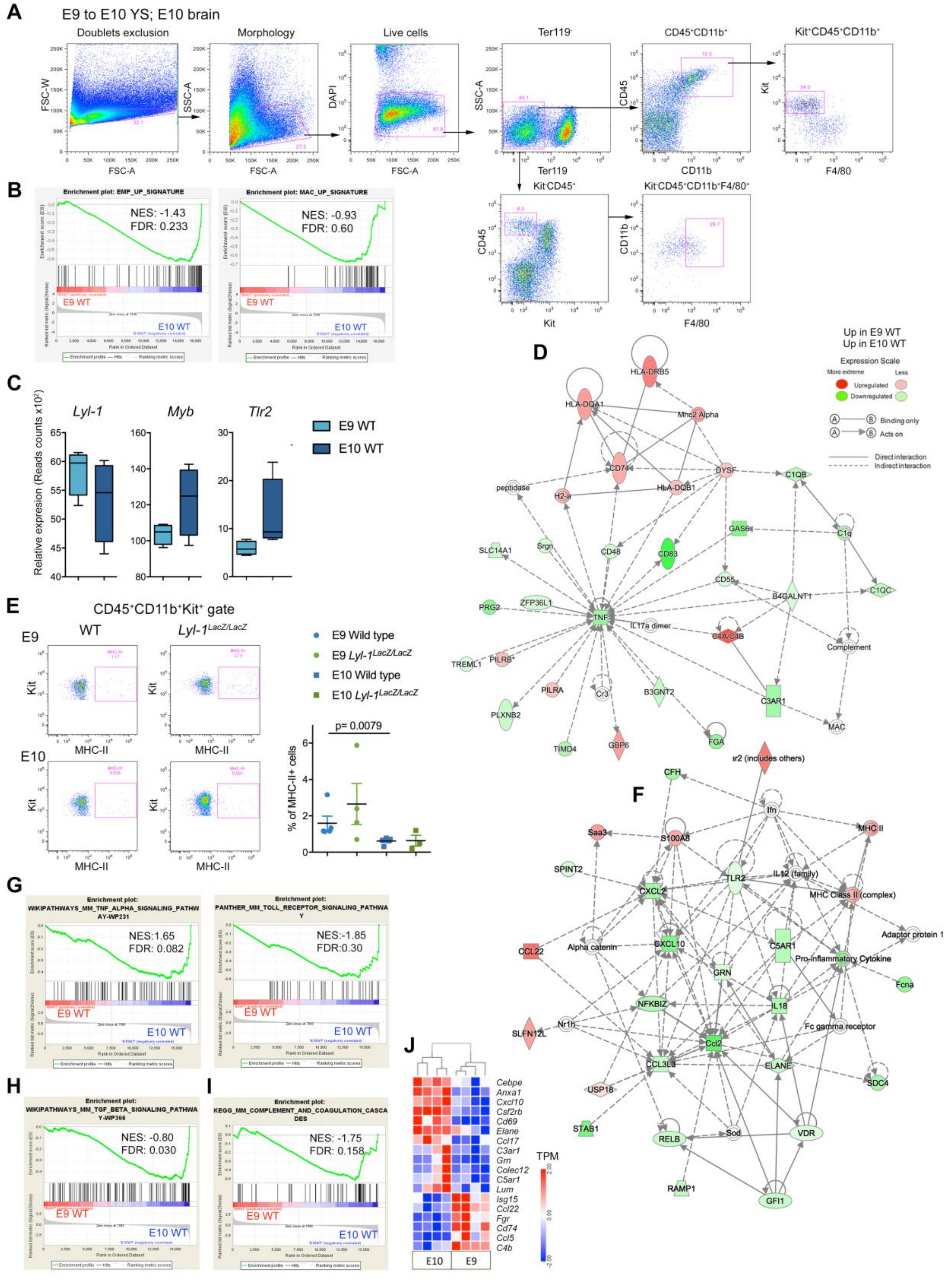

Supplementary figure 1, related to figure 1:

a. Gating strategy used to analyze FDG⁺/Lyl-1⁺ expression in MΦ-progenitors from E9- and E9.5-YS from

WT, *Lyl-1*$^{WT/LacZ}$ and *Lyl-1*$^{LacZ/LacZ}$ embryos ***(Figure 1b)*** and in MΦ-progenitors and mature MΦ-progenitors from E10 WT and *Lyl-1*$^{LacZ/LacZ}$ YS, FL and brain ***(Figure 4a, 5a, c, g)***. Shown is a representative flow cytometry profile of WT YS sample at E10.

b. Gene set enrichment analysis (GSEA) of the whole transcriptome of WT E9-YS MΦ-progenitors, compared to EMP and MΦs signatures defined by Mass *et al.*[2] (NES: normalized enrichment score; FDR: false discovery rate).

c. Relative expression levels of factors thought to characterize either primitive (*Lyl-1*) or transient definitive progenitors (*Myb*, *Tlr2*). A tendency to decrease (*Lyl-1*) or to increase (*cMyb*, *Tlr2*) at E10 is consistent with their wave of origin, albeit at non-significant levels due to the presence of MΦ progenitors from both waves in E10-YS (Box and whisker plots, min to max; median is shown; *p*-values were determined by unpaired, two-tailed *t*-Test).

d. Top 1 Ingenuity Pathway Analyses network representing the molecular relationships between DEG in E9 vs. E10 WT MΦ-progenitors indicated an overexpression of MHC-II at E9, while E10 progenitors over-expressed the TNFα signaling pathway.

e. Flow cytometry analysis of MHC-II expression in MΦ-progenitors from WT E9- and E10-YS. MHC-II expression is significantly reduced in E10 Kit$^+$CD45$^+$CD11b$^+$ MΦ-progenitors compared to E9, irrelevant of the genotype. Representative flow cytometry profile. Quantification of MHC-II expression: (E9 WT: n=5; E9 *Lyl-1*$^{LacZ/LacZ}$: n=4; E10 WT: n=5; E10 E9 *Lyl-1*$^{LacZ/LacZ}$: n=3; Error bars show mean ± s.e.m.; Two tailed, unpaired Mann-Whitney *t*-test).

f. Top 5 Ingenuity Pathway analysis network Illustrates the bias towards inflammatory signaling in E10 WT MΦ-progenitors, compared to those present at E9.

g. Enrichment plot of TLR and TNFα signaling pathways (GSEA pathways): WT MΦ-progenitors at E10 were enriched in TLR and TNFα signaling pathways (NES: Normalized enrichment score; FDR: False discovery rate).

h. Enrichment plot of TGFβ signaling pathway (GSEA pathways). WT MΦ-progenitors at E10 were enriched in TGFβ signaling pathway (NES: Normalized enrichment score; FDR: False discovery rate).

i. Enrichment plot of the complement cascade (KEGG pathway): WT MΦ-progenitors at E10 were enriched in genes belonging to the complement cascade (NES: Normalized enrichment score; FDR: False discovery rate).

j. Expression profiles of DEGs related to phagocytosis (Heatmap displays transformed log2-expression values; unpaired *t*-Test, two-tailed).

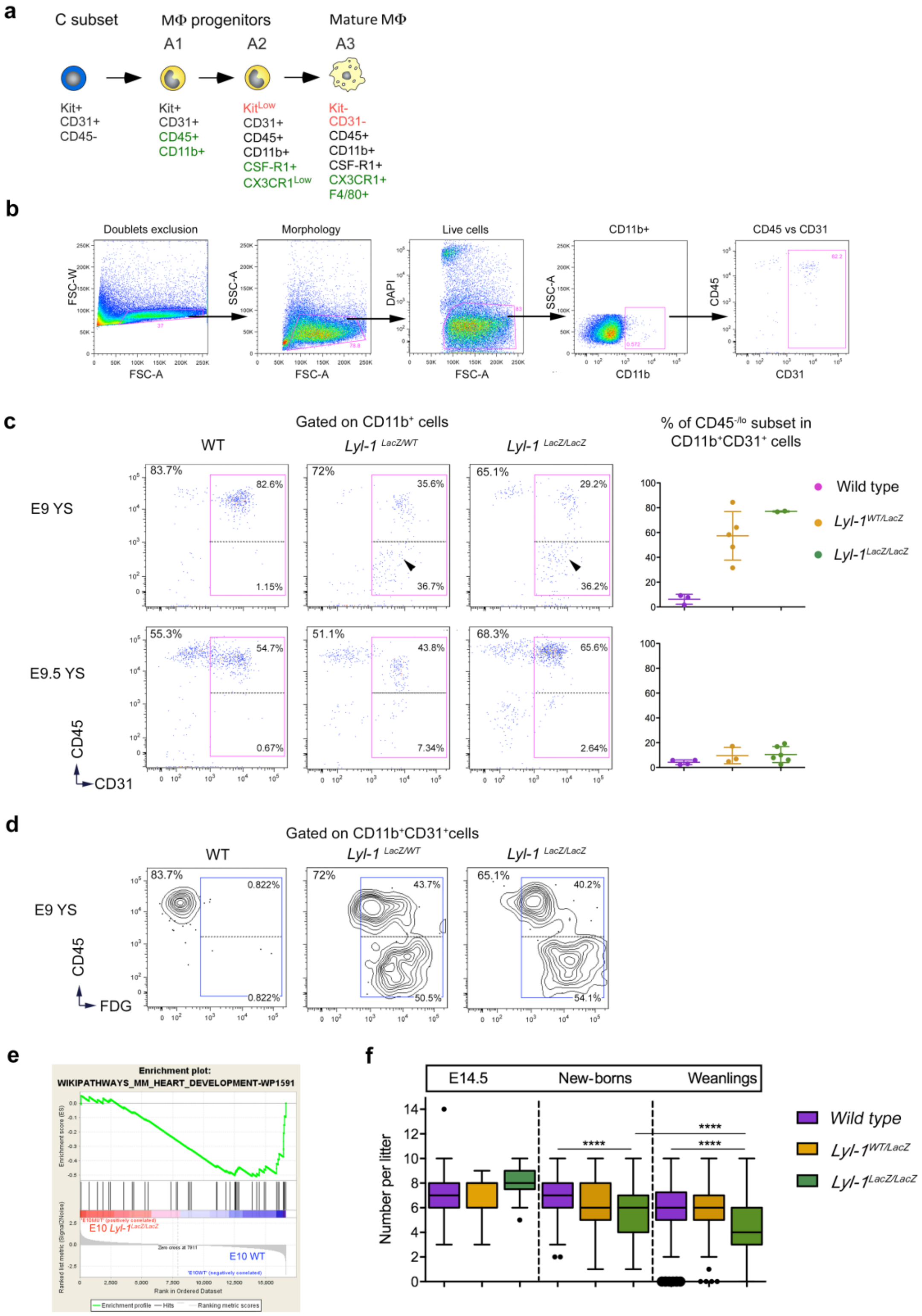

Supplementary figure 2, related to figure 3:

a. Phenotype of MΦ subsets. MΦ develop from $Kit^+CD31^+CD45^-$ progenitors (C subset). MΦ-progenitors first acquire the expression of CD11b and CD45 (A1 subset), then down-regulate Kit expression (A2 subset). The differentiation to mature MΦs is hallmarked by the concomitant down-regulation of CD31 and up-regulation of F4/80 and CX_3CR1 (A3 subset).

b. Gating strategy used to characterize MΦ-progenitor development from E9- and E9.5-YS. Note that this gating strategy differs from the one used to analyze the E9-10 MΦs ***(Supplementary figure 1a)*** in the YS and brain, which lack the $CD11b^+CD31^+CD45^{neg/low}$ intermediate progenitor subset.

c. Lyl-1-deficiency leads to an increased commitment of mesodermal/pre-hematopoietic cells to a MΦ fate. At E9, *Lyl-1*$^{WT/LacZ}$ and *Lyl-1*$^{LacZ/LacZ}$ YS harbor a large $CD45^{neg/low}$ subset within CD11b+ CD31+ cells (arrowhead), which nearly absent from WT YS samples. This C to A1 transition subset is no longer present in mutant YS at E9.5. Representative profiles of independent experiments. Quantification of the C to A1 transition subset in E9 and E9.5-YS (each dot represent an independent experiment; Error bars show mean ± s.e.m.; Two tailed, unpaired *t*-test).

d. Facs-Gal analysis of Lyl-1 expression in E9-YS MΦ-progenitors. Both $CD45^+$ and the C to A1/CD45- subsets within the $CD11b^+$ $CD31^+$ MΦP gate express FDG/Lyl-1. The contour plots in WT samples indicate the level of non-specific β-Gal activity. Representative profiles of 4 independent experiments.

e. Enrichment plot of top 1 GSEA pathway down-regulated in *Lyl-1*$^{LacZ/LacZ}$ MΦ-progenitors compared to WT at E10 is related to heart development (NES: Normalized enrichment score; FDR: False discovery rate).

f. Lyl-1 deficiency leads to an increased perinatal lethality: The number of *Lyl-1*$^{LacZ/LacZ}$ embryos per litter was unmodified at E14.5, but the number of living newborns was reduced. The number of *Lyl-1*$^{LacZ/LacZ}$ pups was further reduced at weaning with a significant increase of neonatal lethality until P15, with a main occurrence between P1 and P5. *Lyl-1*$^{LacZ/LacZ}$ had a balanced sex ratio at birth and weaning. Similar numbers of E14.5 embryos, newborns and weanling were obtained from WT and *Lyl-1*$^{WT/LacZ}$ resulting from WT x *Lyl-1*$^{LacZ/LacZ}$ crosses (E14: n = WT: 41; *Lyl-1*$^{WT/LacZ}$: 39; *Lyl-1*$^{LacZ/LacZ}$: 33. Newborns and weanlings: n = WT: 230; *Lyl-1*$^{WT/LacZ}$: 95; *Lyl-1*$^{LacZ/LacZ}$: 432 (Box and whisker plots, min to max; median is shown; Two tailed, unpaired *t*-test).

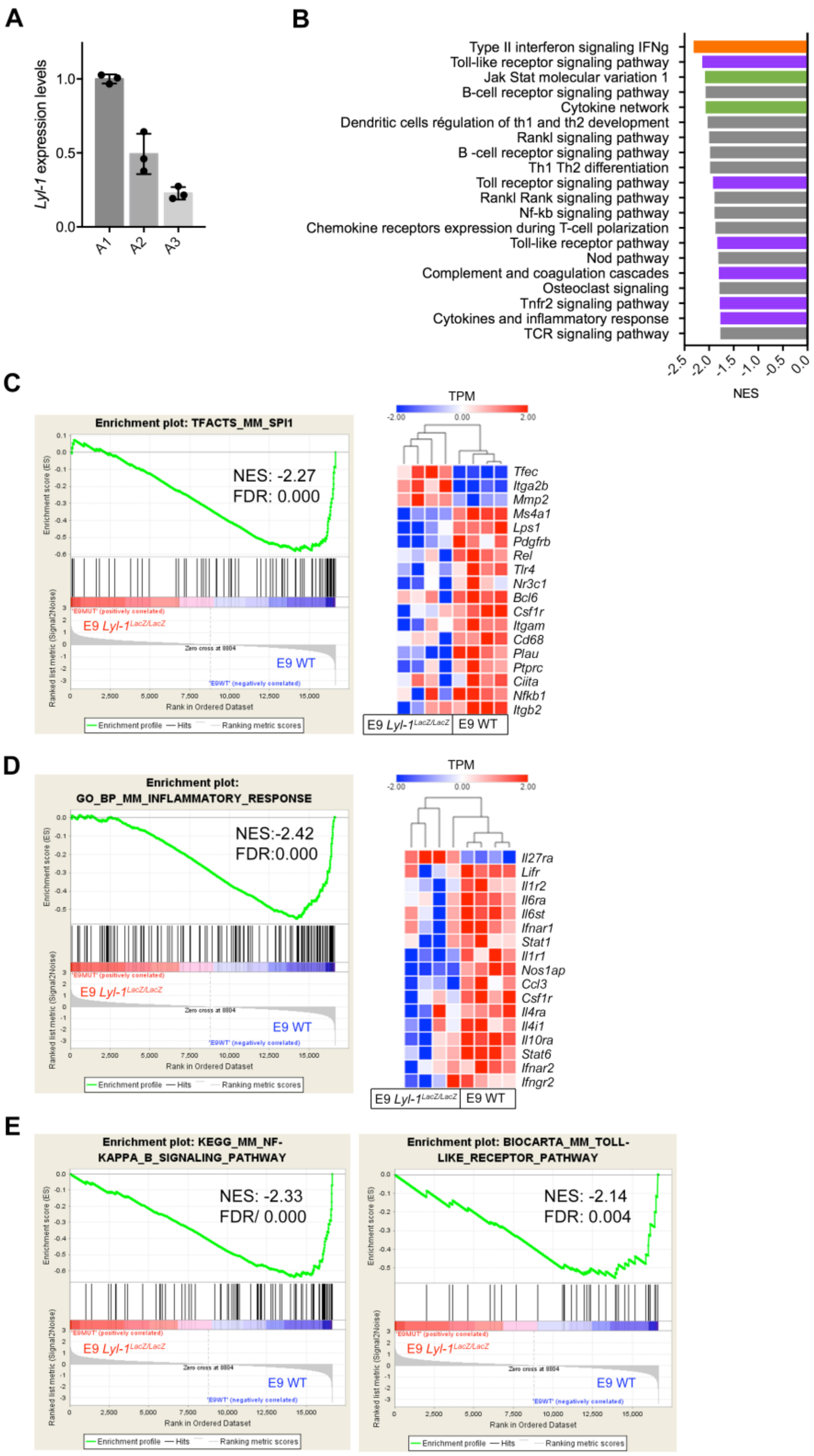

Supplementary figure 3, related to Figure 4

a. RT-qPCR analyses of *Lyl-1* expression in A1 to A3 MΦ subsets from *Cx3cr1*$^{WT/GFP}$ E10-YS. *Lyl-1* is expressed by the 3 subsets with an expression level decreasing upon differentiation. Expression levels were normalized to the mean value obtained for *Cx3cr1*$^{WT/GFP}$ A1 progenitors (n=3).

b. Top GSEA pathways weakened in *Lyl-1*$^{LacZ/LacZ}$ compared to WT MΦ-progenitors at E9 (FDR q-value <0.05). Highlighted are pathways related to cytokine signaling (green), pathways enriched in E9 WT compared to E10 WT that are down-regulated due to *Lyl-1*-deficiency (orange) and the pathways enriched in E10 WT compared to E9 WT that were further reduced in *Lyl-1*$^{LacZ/LacZ}$ MΦ-progenitors at E9 (purple).

c. Enrichment plot of the *Spi1*/PU.1 pathway (Top 2 GSEA transcription factor pathway): *Spi1*/PU.1 signaling was weaker in E9 *Lyl-1*$^{LacZ/LacZ}$ than in WT E9 MΦ-progenitors (NES: normalized enrichment score; FDR: false discovery rate). Expression profiles of DEGs related to *Spi1*/PU.1 pathway (Heatmap displays transformed log2-expression values; unpaired t-Test, two-tailed).

d. Enrichment plot of the inflammatory response (Top 1 GO enrichment plot): The inflammatory response is defective in E9 *Lyl-1*$^{LacZ/LacZ}$ MΦ-progenitors (NES: normalized enrichment score; FDR: false discovery rate). Expression profiles of DEGs related to inflammatory response and cytokine signaling. (Heatmap displays transformed log2-expression values; Unpaired *t*-Test, two-tailed).

e. Enrichment plots of NFkB (Top1 KEGG pathway) and Toll-like receptor (Top 2 GSEA pathway): NFkB and Toll-like receptor signaling were weaker in E9 *Lyl-1*$^{LacZ/LacZ}$, compared to WT E9 MΦ-progenitors (NES: normalized enrichment score; FDR: false discovery rate).

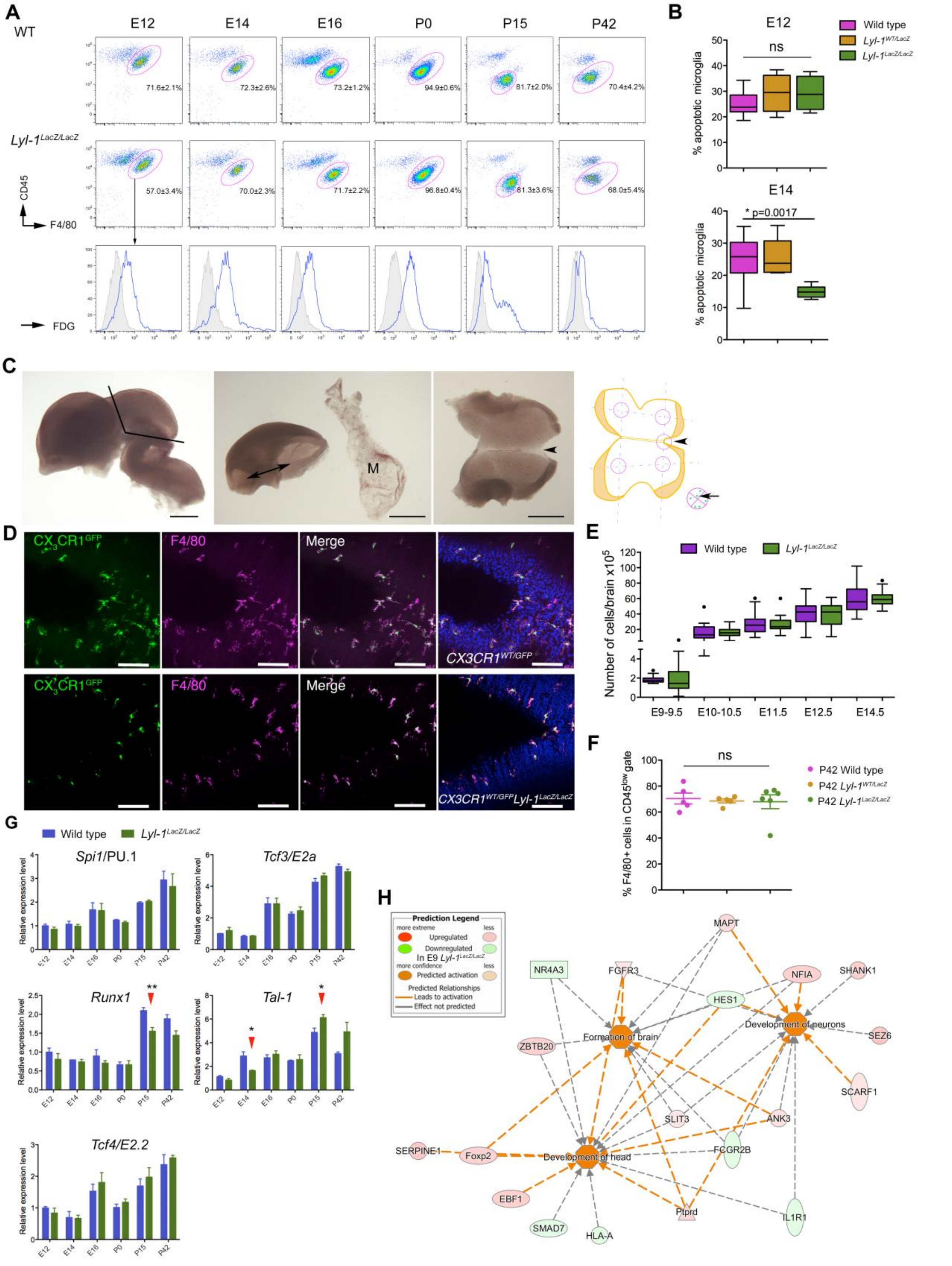

Supplementary figure 4, related to figure 6

a. Microglia express Lyl-1 from embryonic stages to adulthood. FDG/Lyl expression in WT and *Lyl-1*$^{LacZ/LacZ}$ brain from E12 to adult stages. In *Lyl-1*$^{LacZ/LacZ}$ mutants, the entire microglia population is FDG^{+}/Lyl-1^{+}, but

Lyl-1 expression level decreases in the adult, with a clear transition occurring at P15. Plain grey histograms indicate non-specific background β-Gal activity/FDG levels in WT samples.

b. Annexin V-7AAD quantification of apoptosis levels in $CD11b^+F4/80^+CD45^{low}$ microglia at E12 and E14. No significant genotype-associated modification of microglia apoptosis level was observed in E12 microglia (WT: n=6; *Lyl-1*$^{WT/LacZ}$ and *Lyl-1*$^{LacZ/LacZ}$: n=4). At E14 (WT, *Lyl-1*$^{WT/LacZ}$ and *Lyl-1*$^{LacZ/LacZ}$: n=6), the apoptosis level was significantly lower in *Lyl-1*$^{LacZ/LacZ}$ compared to WT and *Lyl-1*$^{WT/LacZ}$ brains, which may account for the recovery of microglia pool size after E12. (Box and whisker plots, min to max; median is shown; unpaired *t*-Test, two-tailed).

c. The midbrain was dissected from *Cx3cr1*$^{WT/GFP}$*:Lyl-1*$^{WT/WT}$ and *Cx3cr1*$^{WT/GFP}$*:Lyl-1*$^{LacZ/LacZ}$ embryos. After removal of the meningeal layer (M), the midbrain was sectioned along the midline and flat mounted. To unsure an unbiased choice, cells were always acquired in the same location using the midline (arrowhead) and lateral thickening as landmarks: the cell closest to the middle of the frame (arrow) was acquired (Bar=1mm).

d. Confocal imaging of the midbrain of *Cx3cr1*$^{WT/GFP}$ and *Cx3cr1*$^{WT/GFP}$*:Lyl-1*$^{LacZ/LacZ}$ embryos at E12 pointed to *Lyl-1*$^{LacZ/LacZ}$-induced morphological changes in microglia. Microglia were identified by *Cx3cr1*-driven GFP expression and F4/80 immunostaining (Bar=100 μm).

e. Evolution of brain cellularity from E9 to E14.5 in WT and *Lyl-1*$^{LacZ/LacZ}$ embryos: 29 to 40 individual brain suspensions were counted in 10 (E9-9.5 and E10-10.5), 6 (E11.5), 8 (E12.5) and 7 (E14.5) individual experiments (Box and whisker plots, min to max; median is shown; unpaired *t*-Test, two-tailed). *Lyl-1*-deficiency does not lead to modifications of cells recovery from E9 to E14 whole brains.

f. Cytometry analyses evidenced no genotype-associated difference in microglia pool size in the brain from adult WT, *Lyl-1*$^{WT/LacZ}$ and *Lyl-1*$^{LacZ/LacZ}$ mice. (3 independent experiments. Error bars show mean $\pm$ s.e.m.; unpaired *t*-Test, two-tailed).

g. $CD11b^+F4/80^+CD45^{low}$ microglia was isolated at sequential development stages for quantitative RT-PCR analyses. Bar graphs show the kinetic of expression of a selected set of genes that were not significantly modified in *Lyl-1* mutants at E12 and P0-P3, but may be so at post-natal stages. Gene expressions were normalized to the mean expression value in WT E12 microglia (n=3). Floating bars indicate mean $\pm$ s.e.m. (*P* values were determined by unpaired, two-tailed *t*-Test).

h. IPA function prediction: A function in neurogenesis is predicted for E9 *Lyl-1*$^{LacZ/LacZ}$ MΦ-progenitors considering the DEGs enriched compared to E9 WT.

Supplementary tables

Supplementary table 1: GSEA gene sets enriched in WT MΦP at E10

Compared to E9 MΦ progenitors, E10 progenitors were enriched in pathways involved in the regulation of erythro-myeloid lineages (Green), in inflammatory signaling and in metabolism (pink).

Rank	Gene sets enriched in E10 WT compared to E9 WT MΦ progenitors	Size	NES	Nom.p-value	FDR q-val
1	Pyruvate metabolism	34	-1.98	0.0	0.027
2	Diurnally regulated genes with circadian orthologs	40	-1.93	0.0	0.039
3	Butanoate metabolism	16	-1.90	0.0	0.040
4	Epo signaling pathway	15	-1.88	0.0	0.045
5	Oxidative stress response	45	-1.87	0.0	0.038
6	ATM signaling pathway	17	-1.86	0.0032	0.034
7	Blood coagulation	37	-1.86	0.0	0.030
8	Toll receptor signaling pathway	38	-1.85	0.0	0.031
9	Adipogenesis	125	-1.85	0.0	0.028
10	Estrogen signaling pathway	18	-1.85	0.0	0.026
11	TNF-R2 signaling pathway	17	-1.81	0.0	0.038
12	DNA damage response only ATM dependent	77	-1.81	0.0028	0.036
13	TGFβ signaling pathway	109	-1.80	0.0	0.034
14	Alpha6 beta4 integrin	69	-1.80	0.0	0.036
15	Propanoate metabolism	23	-1.78	0.0032	0.040
16	TPO signaling pathway	18	-1.78	0.0	0.039
17	Folate metabolism	46	-1.77	0.0029	0.040
18	Gene regulation by peroxisome proliferators via ppara-alpha	42	-1.77	0.0	0.038
19	Oxidative stress induced gene expression via NRF2	16	-1.76	0.0033	0.039
20	Steroids metabolism	15	-1.74	0.0016	0.047
21	TGFβ signaling pathway (wp560)	51	-1.74	0.0013	0.045
22	Il-3 signaling pathway	39	-1.74	0.0014	0.046
23	Mets effect on macrophage differentiation	15	-1.73	0.006600	0.049

Supplementary table 2: ***GSEA pathways related to developmental patterning enriched in E9 Lyl-1$^{LacZ/LacZ}$ MΦ-progenitors.***

Rank	GSEA Pathway up in E9 Mut vs WT *MΦ* progenitors	SIZE	NES	NOM p-val	FDR q-val	
4	Cadherin signaling pathway	97	1.90	0.000	0.022	*Cdh3; Cdh5; Wnt3; Pcdhb6; Erbb4; Wnt7b; Wnt1; Tcf7l1; Pcdh8; Cdh20; Wnt4; Cdh9; Pcdh19; Ctnna3; Wnt7a; Cdh8; Pcdha4; Wnt9a; Pcdh18; Wnt5a; Cdh6; Src; Cdh18; Erbb3; Cdh4; Erbb2; Cdh24; Fzd9; Celsr2; Fzd8; Pcdhb4; Cdh11; Celsr1; Acta2; Wnt2b; Wnt11; Pcdhb5; Cdh7; Fat3*
12	Cytoskeletal regulation by RHO GTPase	60	1.57	0.013	0.242	*Myh3; Myh1; Rhoj; Pak6; Myo3a; Rhoc; Myh6; Myh14; Myh2; Tubb6; Stmn4; Acta2; Myh7; Actg2; Pak3; Vasp; Pak4; Actbl2; Myh4; Arpc4; Rac2; Pak1*
13	Wnt signaling pathway	225	1.55	0.002	0.247	*Myh3; Myh1; Nkd1; Celsr3; Sfrp5; Cdh3; Cdhr1; Wnt3; Pcdhb6; Wnt7b; Wnt1; Tcf7l1; Pcdh8; Dkk4; Cdh20; Sfrp2; Wnt4; Cdh9; Myh6; Mycn; Pcdh19; Ctnna3; Axin2; Wnt7a; Cdh8; Pcdha4; Wnt9a; Pcdh18; Acvr1b; Myh2; Wnt5a; Prkcz; Cdh6; Smarcd3; Pygo1; Edn1; Tle2; Cdh18; Cdh4; Gng13; Cdh24; Arr3; Fzd9; Celsr2; Fzd8; Pcdhb4; Cdh11; Celsr1; Gng12; Gna11; En1; Ctnnal1; Acta2*
25	Integrin signaling pathway	162	1.41	0.013	0.340	*Itga2b; Lama4; Col17a1; Col11a2; Lamb2; Col8a2; Parvb; Itgae; Col18a1; Col19a1; Col5a3; Rhoc; Col9a3; Rnd2; Parva; Itga1; Col4a2; Rnd1; Col5a1; Col6a1; Pik3c2g; Grap; Col11a1; Col1a1; Col6a2; Col4a1; Col3a1; Src; Col1a2; Col12a1; Col9a1; Itgbl1; Itgb3; Mapk3; Lamc2; Col10a1; Col4a6; Lims2; Map2k3; Itga8; Col4a3*

Supplementary table 3: Lyl-1-positive MΦ^{Prim} progenitors from E10-YS could play a role in heart development and function. KEGG gene sets down-regulated in E10 MΦ progenitors in *Lyl-1$^{LacZ/LacZ}$*, compared to WT.

Rank	Gene sets	SIZE	NES	NOM p-val	FDR q-val
1	Myl3 pathway (Reactome mm)	18	-2.08	0.000	0.004
2	Myl1 pathway (Reactome mm)	20	-2.08	0.000	0.003
3	Tcap pathway (Reactome mm) (Titin, muscle)	15	-2.04	0.000	0.004
4	Neb pathway (Reactome mm) (Nebulin, muscle, myopathy)	16	-2.00	0.000	0.006
5	Myl4 pathway (Reactome mm)	17	-1.99	0.000	0.005
6	Myh8 pathway (Reactome mm)	21	-1.94	0.000	0.011
7	Myh6 pathway (Reactome mm)	21	-1.93	0.000	0.012
8	Myh3 pathway (Reactome mm)	22	-1.92	0.000	0.012
9	Mybpc3 pathway (Reactome mm)	23	-1.87	0.000	0.025
10	Mybpc2 pathway (Reactome mm)	23	-1.87	0.000	0.023
11	Mybpc1 pathway (Reactome mm)	23	-1.87	0.000	0.021
12	Myl2 pathway (Reactome mm)	28	-1.82	0.000	0.047
13	Desmin pathway (Reactome mm) (Muscle-cardiopathy)	27	-1.77	0.000	0.092
14	Antigen processing and presentation (Kegg mm)	34	-1.65	0.005	0.386
15	Ncor1 pathway (Reactome mm)	44	-1.65	0.006	0.364
16	Dmd (dystrophin) pathway (Reactome mm)	51	-1.62	0.009	0.442
17	Hmgb1 pathway (Reactome mm)	24	-1.61	0.016	0.482
18	Ap2m1 pathway (Reactome mm)	24	-1.61	0.003	0.468
19	Fgf1_pathway (Reactome mm)	30	-1.60	0.011	0.478
20	Cytokine-cytokine receptor interaction (Kegg mm)	199	-1.58	0.002	0.535

Supplementary table 4: GSEA transcription factors

Differential expression of transcription factor gene sets between E9 and E10 MΦ progenitors in a WT context.

Rank	Gene set enriched in WT MΦ progenitors at E9	SIZE	NES	NOM p-val	FDR q-val
1	REST (TFACTS-MM)	19	1.68	0.010	0.182
**Rank**	**Gene sets enriched in WT MΦ progenitors at E10**	**SIZE**	**NES**	**NOM p-val**	**FDR q-val**
1	GATA1 (TFACTS-MM)	23	-2.53	0.000	0.000
2	ESR1 (TFACTS-MM)	47	-1.95	0.000	0.015
3	JUN (TF-MM-Zhao)	93	-1.94	0.000	0.011
4	JUND (TFACTS-MM)	24	-1.92	0.000	0.011
5	CEBPB (TF-MM-Zhao)	20	-1.92	0.000	0.009
6	RARA (TFACTS-MM)	51	-1.91	0.000	0.008
7	JUN (TFACTS-MM)	103	-1.90	0.000	0.009
8	SREBF1 (TFACTS-MM)	44	-1.90	0.000	0.008
9	JUNB (TFACTS-MM)	15	-1.89	0.000	0.007
10	SFPI1 (TF-MM-Zhao)	41	-1.89	0.000	0.007
11	SMAD7 (TFACTS-MM)	15	-1.88	0.000	0.008
12	SREBF2 (TFACTS-MM)	31	-1.87	0.001	0.008
13	LEF1 (TFACTS-MM)	43	-1.84	0.001	0.011
14	YY1 (TFACTS-MM)	37	-1.84	0.002	0.011
15	SP1 (TFACTS-MM)	352	-1.82	0.000	0.012
16	SMAD4 (TFACTS-MM)	38	-1.82	0.005	0.012
17	ETS1 (TFACTS-MM)	114	-1.79	0.001	0.014
18	ELK1 (TFACTS-MM)	15	-1.79	0.000	0.014
19	AR (TFACTS-MM)	45	-1.78	0.001	0.015
20	SMAD3 (TFACTS-MM)	53	-1.78	0.000	0.014
21	ATF2 (TFACTS-MM)	28	-1.75	0.005	0.020
22	FOXO1 (TFACTS-MM)	135	-1.74	0.000	0.021
23	HOXC8 (TF-MM-Zhao)	25	-1.72	0.008	0.025
24	RXRA (TFACTS-MM)	20	-1.72	0.003	0.024

Supplementary table 5: GSEA transcription factors

Transcription factor gene sets modified in E9 *Lyl-1*$^{LacZ/LacZ}$ MΦ progenitors, compared to WT.

Rank	Gene set enriched in E9 ***Lyl-1***$^{LacZ/LacZ}$ MΦ progenitors	SIZE	NES	NOM p-val	FDR q-val
1	SMAD1 (TF-MM-Zhao)	15	1.66	0.024	0.363
**Rank**	**Gene sets depleted in E9 *Lyl-1*$^{LacZ/LacZ}$ MΦ progenitors**	**SIZE**	**NES**	**NOM p-val**	**FDR q-val**
1	SFPI1 (TF-MM)	41	-0.74	0.000	0.000
2	SPI1 (TFACTS-MM)	73	-0.58	0.000	0.000
3	STAT1 (TFACTS-MM)	42	-0.62	0.000	0.000
4	JUN (TF-MM-Zhao)	93	-0.49	0.000	0.004
5	PPARG (TF-MM-Zhao)	37	-0.56	0.000	0.019
6	TBP (TF-MM-Friard)	39	-0.53	0.000	0.049
7	SFPI1 (TF-MM)	29	-0.56	0.000	0.043
8	JUN (TFACTS-MM)	103	-0.43	0.000	0.046
9	NFKB1 (TF-MM-Zhao)	65	-0.46	0.002	0.047
10	NFKB1 (TFACTS-MM)	111	-0.42	0.000	0.045
11	CEBPA (TFACTS-MM)	93	-0.43	0.000	0.046

Supplementary references

1. Bertrand JY, Jalil A, Klaine M, Jung S, Cumano A, Godin I. Three pathways to mature macrophages in the early mouse yolk sac. *Blood*. 2005;106(9):3004-3011.

2. Mass E, Ballesteros I, Farlik M, et al. Specification of tissue-resident macrophages during organogenesis. *Science*. 2016;353(6304).
