# Supplementary material for: Lyl-1 regulates primitive macrophages and microglia development
Source: Commun Biol. 2021 Dec 9;4:1382. doi: 10.1038/s42003-021-02886-5 (PMC8660792; doi:10.1038/s42003-021-02886-5)
Supplement: Supplementary file 3 — Description of Additional Supplementary Files [file 42003_2021_2886_MOESM3_ESM.pdf]

Description of Additional Supplementary Files

File name: Supplementary Data 1.
Description: Source data for figures 1 to 6 and Supplementary figures 1 to 4.
