# Supplementary material for: Lyl-1 regulates primitive macrophages and microglia development
Source: Commun Biol. 2021 Dec 9;4:1382. doi: 10.1038/s42003-021-02886-5 (PMC8660792; doi:10.1038/s42003-021-02886-5)
Supplement: Supplementary file 5 — Reporting Summary [file 42003_2021_2886_MOESM5_ESM.pdf]

nature research

Corresponding author(s): GODIN Isabelle
Last updated by author(s): Oct 27, 2021

Reporting Summary

Nature Research wishes to improve the reproducibility of the work that we publish. This form provides structure for consistency and transparency in reporting. For further information on Nature Research policies, see our Editorial Policies and the Editorial Policy Checklist.

Statistics

For all statistical analyses, confirm that the following items are present in the figure legend, table legend, main text, or Methods section.

n/a	Confirmed	
☐	☒	The exact sample size (*n*) for each experimental group/condition, given as a discrete number and unit of measurement
☐	☒	A statement on whether measurements were taken from distinct samples or whether the same sample was measured repeatedly
☐	☒	The statistical test(s) used AND whether they are one- or two-sided<br>*Only common tests should be described solely by name; describe more complex techniques in the Methods section.*
☒	☐	A description of all covariates tested
☒	☐	A description of any assumptions or corrections, such as tests of normality and adjustment for multiple comparisons
☐	☒	A full description of the statistical parameters including central tendency (e.g. means) or other basic estimates (e.g. regression coefficient) AND variation (e.g. standard deviation) or associated estimates of uncertainty (e.g. confidence intervals)
☐	☒	For null hypothesis testing, the test statistic (e.g. *F*, *t*, *r*) with confidence intervals, effect sizes, degrees of freedom and *P* value noted<br>*Give P values as exact values whenever suitable.*
☒	☐	For Bayesian analysis, information on the choice of priors and Markov chain Monte Carlo settings
☒	☐	For hierarchical and complex designs, identification of the appropriate level for tests and full reporting of outcomes
☒	☐	Estimates of effect sizes (e.g. Cohen's *d*, Pearson's *r*), indicating how they were calculated

Our web collection on statistics for biologists contains articles on many of the points above.

Software and code

Policy information about availability of computer code

Data collection	For confocal imaging, image stacks were collected using a Leica SP8 confocal microscope. For RNA-seq., libraries were bar-coded, purified, pooled together in equal concentrations and subjected to paired-end sequencing (2x100) on Novaseq-6000 sequencer (Illumina) at the Gustave Roussy genomic facility.
Data analysis	Data were analysed using GraphPad Prism 7 for all statistical analyses, Flowjo10.0.7 (Tree Star, Ashland, OR) for flow cytometry analyses, Photoshop 8.0.1 (Adobe) and Imaris software (Bitplane) for analysis of imaging data. RNA-seq data were analyzed using Ingenuity® Pathway Analysis (IPA, QIAGEN), Gene set enrichment analysis (GSEA), Morpheus and Venny softwares.

For manuscripts utilizing custom algorithms or software that are central to the research but not yet described in published literature, software must be made available to editors and reviewers. We strongly encourage code deposition in a community repository (e.g. GitHub). See the Nature Research guidelines for submitting code & software for further information.

Data

Policy information about availability of data

All manuscripts must include a data availability statement. This statement should provide the following information, where applicable:

- Accession codes, unique identifiers, or web links for publicly available datasets
- A list of figures that have associated raw data
- A description of any restrictions on data availability

RNA-seq. data (accession number E-MTAB-9618) were deposited in EMBL-EBI ArrayExpress database (www.ebi.ac.uk/arrayexpress)

Field-specific reporting

Please select the one below that is the best fit for your research. If you are not sure, read the appropriate sections before making your selection.

☒ Life sciences ☐ Behavioural & social sciences ☐ Ecological, evolutionary & environmental sciences

For a reference copy of the document with all sections, see nature.com/documents/nr-reporting-summary-flat.pdf

Life sciences study design

All studies must disclose on these points even when the disclosure is negative.

Sample size	No statistical methods were used to predetermine sample size.
Data exclusions	Low quality samples such as sample with high cell death percentage were removed from the analysis.
Replication	Replication of experiments set were successfull
Randomization	No randomization method was used. Animals were allocated into experimental groups according to genotype.<br>For the morphology analysis of E12 microglia, the imaged cells were selected according to the morphological landmarks described in Supplemental Figure 4c to ensure an unbiaised choice.
Blinding	No blinding was used for the collection of embryonic tissues (known genotype).

Reporting for specific materials, systems and methods

We require information from authors about some types of materials, experimental systems and methods used in many studies. Here, indicate whether each material, system or method listed is relevant to your study. If you are not sure if a list item applies to your research, read the appropriate section before selecting a response.

Materials & experimental systems

n/a	Involved in the study	
☐	☒	Antibodies
☒	☐	Eukaryotic cell lines
☒	☐	Palaeontology and archaeology
☐	☒	Animals and other organisms
☒	☐	Human research participants
☒	☐	Clinical data
☒	☐	Dual use research of concern

Methods

n/a	Involved in the study	
☒	☐	ChIP-seq
☐	☒	Flow cytometry
☒	☐	MRI-based neuroimaging

Antibodies

Antibodies used	Ter119 (clone TER-119) FITC: Biolegend 116205; PE: Biolegend 116208; APC eBioscience 17-5921-82<br>F4/80 (clone BM8) Alexa fluor 488: eBioscience 53-4801-82; FITC: Biolegend 122606; PE: eBioscience 12-4801-82; APC: Biolegend 123116<br>GR-1 (Clone RB6-8C5) FITC: BD-Pharmingen 553127; PE-Cy7: Biolegend 108416; BV510: Biolegend 108437<br>CD45 (clone 30-F11) PE: BD-Pharmingen 553081; PE-Cy5: BD-Pharmingen 553082; PE-Cy7: eBioscience 25-0451-82; Alexa fluor 647: Biolegend 103124<br>CD31 (Clone390) PE: Biolegend 102408; (Clone MEC13.3) Alexa fluor 488: Biolegend 102514; PE-Cy5.5: Biolegend 102419; Alexa fluor 647: Biolegend 102516; BV510: BD-Pharmingen 583089<br>Kit (Clone 2B8) Alexa fluor 488: Biolegend; 05824; APC: BD-Pharmingen; 553358; APC-Cy7: Biolegend 105825<br>CD11b (Clone M1/70) PE-Cy5.5: eBioscience 45-0112-82; PE-Cy7: Biolegend 101215; APC: Biolegend 101212; APC-eFluor 780: eBioscience 47-0112-82<br>Sca-1 (Clone D7) PE-Cy7: Biolegend; 108113<br>MHC-II (Clone M5/114.15.2) PE: Biolegend 107607<br>Anti-Brdu (Clone B44) APC: BD-Pharmingen 51-23619L
Validation	All antibodies were titrated and used based on manufacturer recommendations

Animals and other organisms

Policy information about studies involving animals; ARRIVE guidelines recommended for reporting animal research

Laboratory animals	1- C57BL/6 from Harlan or Charles Rivers Laboratories, France (males: 2-18 months; females: 2-8 months)<br>2- Lyl-1LacZ/LacZ (males: 2-18 months; females: 2-8 months)<br>3- Cx3cr1GFP/GFP (males: 2-18 months; females: 2-8 months)<br>4- Cx3cr1GFP/GFP:Lyl-1LacZ/LacZ (males: 2-18 months; females: 2-8 months)<br>Females were mated with males to obtained embryos at various development stages of timed pregancies.
Wild animals	The study did not involve wild animals
Field-collected samples	The study did not involve samples collected from the fields.
Ethics oversight	Experiments were conducted in compliance with French/European laws, under authorized project #2016-030-5798, approved by officially accredited local institutional animal (committee n°26) and French “Ministère de la Recherche” ethics boards.

Note that full information on the approval of the study protocol must also be provided in the manuscript.

Flow Cytometry

Plots

Confirm that:

☒ The axis labels state the marker and fluorochrome used (e.g. CD4-FITC).

☒ The axis scales are clearly visible. Include numbers along axes only for bottom left plot of group (a 'group' is an analysis of identical markers).

☒ All plots are contour plots with outliers or pseudocolor plots.

☒ A numerical value for number of cells or percentage (with statistics) is provided.

Methodology

Sample preparation	Cells from the yolk sac (E7.5-E10.5) or whole brain (E9-E14) were obtained after mechanical disruption. From E12 to adult stages, microglia were recovered following Percoll (P1644, Sigma) separation. Cells were filtered through a 70 µm cell strainer and centrifuged at 300g for 10 min. Cells were resuspended in Fc block BD solution and incubated at room temperature for 10 minutes. After washing in PBS+10% FCS and centrifugation, cells were resuspended in 50µl PBS + 10%FCS containing the fluorochrome coupled antibodies and incubated at 4°C for 20 minutes. After washing in PBS+10%FCS and centrifugation, cells were resuspended in 50ul PBS+ 10% FCS. Dead cells were excluded by adding 1µg/mL DAPI (Sigma) before acquisition.
Instrument	Cells were acquired (Canto II) or sorted (FACS-Aria III or Influx, BD Biosciences)
Software	Data acquisitions were performed using BD Facs Diva 6.1.3. Data were analysed using Flowjo 10.0.7 (Tree Star, Ashland, OR).
Cell population abundance	The purity for sorted cell samples was not assessed by re-sorting, since populations from early embryos contain low number of cells. Similar sorts done with the same cell types at older stages (e.g. Foetal liver), when cells are more abundant pointed to a cell purity over 95%. Similarly, adult microglia was sorted as 1000 cells samples for RNA-seq experiments. When the whole microglia is sorted and re-analyzed, the purity was over 99%.
Gating strategy	the gating stategies are described in supplemental data 1A and 2B.

☒ Tick this box to confirm that a figure exemplifying the gating strategy is provided in the Supplementary Information.
